# Diagnostic Classification and Biomarker Identification of Alzheimer’s Disease with Random Forest Algorithm †

**DOI:** 10.3390/brainsci11040453

**Published:** 2021-04-02

**Authors:** Minseok Song, Hyeyoom Jung, Seungyong Lee, Donghyeon Kim, Minkyu Ahn

**Affiliations:** 1 School of Computer Science and Electrical Engineering, Handong Global University, Pohang-si 37554, Korea; Minseok.H.Song@gmail.com (M.S.); hyeyoomj@naver.com (H.J.); strikerlee95@gmail.com (S.L.); 2 Neurophet Inc., Gangnam-gu, Seoul 08380, Korea; donghyeon.kim@neurophet.com

**Keywords:** Alzheimer’s disease, mild-cognitive impairment, magnetic resonance imaging, machine learning, Random Forest, feature importance, Gini index, convolutional neural network

## Abstract

Random Forest (RF) is a bagging ensemble model and has many important advantages, such as robustness to noise, an effective structure for complex multimodal data and parallel computing, and also provides important features that help investigate biomarkers. Despite these benefits, RF is not used actively to predict Alzheimer’s disease (AD) with brain MRIs. Recent studies have reported RF’s effectiveness in predicting AD, but the test sample sizes were too small to draw any solid conclusions. Thus, it is timely to compare RF with other learning model methods, including deep learning, particularly with large amounts of data. In this study, we tested RF and various machine learning models with regional volumes from 2250 brain MRIs: 687 normal controls (NC), 1094 mild cognitive impairment (MCI), and 469 AD that ADNI (Alzheimer’s Disease Neuroimaging Initiative database) provided. Three types of features sets (63, 29, and 22 features) were selected, and classification accuracies were computed with RF, Support vector machine (SVM), Multi-layer perceptron (MLP), and Convolutional neural network (CNN). As a result, RF, MLP, and CNN showed high performances of 90.2%, 89.6%, and 90.5% with 63 features. Interestingly, when 22 features were used, RF showed the smallest decrease in accuracy, −3.8%, and the standard deviation did not change significantly, while MLP and CNN yielded decreases in accuracy of −6.8% and −4.5% with changes in the standard deviation from 3.3% to 4.0% for MLP and 2.1% to 7.0% for CNN, indicating that RF predicts AD more reliably with fewer features. In addition, we investigated the importance of the features that RF provides, and identified the hippocampus, amygdala, and inferior lateral ventricle as the major contributors in classifying NC, MCI, and AD. On average, AD showed smaller hippocampus and amygdala volumes and a larger volume of inferior lateral ventricle than those of MCI and NC.

## 1. Introduction

Alzheimer’s disease (AD), a type of dementia, is a neurodegenerative disease that destroys neuronal cells selectively. As the number of patients continues to increase steadily, the disease is emerging as a global problem today because some cases cause death [1]. There are many hypotheses about AD’s pathway and many drugs have been developed to slow or stop the disease’s rate of progression [2]. However, because there are no drugs or treatments that cure AD clearly, early and precise diagnosis of AD is even more critical. One way to diagnose dementia or AD is to use a survey-based test, which include the Clinical Dementia Rating (CDR), Mini-Mental State Exam (MMSE), etc. [3,4]. These are designed to test brain functions, including memory and emotion, to assess the disease’s progression. Surveys are effective when the patients begin to experience symptoms, but it is difficult to identify signs of the disease before symptom onset.

Mild Cognitive Impairment, referred to as MCI, is the early stage of AD. Patients with MCI differ from others in their same age group, but are not affected significantly in their daily lives. Occasionally they experience amnesia or depression, but most are unaware of, or fail to acknowledge that they have the disease. Consequently, more than half of MCI patients progress to dementia within five years [5], and are classified as having AD, which is the most common form of dementia. It alters parts of the brain in many respects. Current well-known biological biomarkers are tau and amyloid-β deposits that affect loss of brain volume or neurons in the hippocampus and cerebral cortex [6]. According to one study, more than 80% of AD and 90% of those with normal cognitive function can be distinguished with total-tau (T-tau), phospho-tau (P-tau), and amyloid-β, [7]. However, diagnosis with these biological biomarkers is too difficult for non-domestic people and is time consuming.

There have been attempts to diagnose the MCI and AD using noninvasive methods. These studies used microRNAs with blood serum [8], pupil dilation response [9], and electroencephalogram [10,11,12,13,14]. Magnetic resonance imaging (MRI), which calculates each brain region’s volume, is also an effective way to diagnose AD. When researchers gained access to MRI data, studies of AD using brain volume began to be conducted actively. Since then, new observable structural biomarkers, such as shrinkage of the hippocampus, have been identified. However, there are some problems using MRI as well. It is difficult to diagnose AD with just one MRI, because reduction in brain volume needs to be observed to do so, except in serious cases. Thus, patients should have an MRI at least every six months to determine differences in the brain’s volume, which is costly and time consuming. Due to this problem, diagnosing AD with only one MRI has become a severe problem. There have been many attempts to interpret images of the brain and more complex models are needed because of the many factors that affect AD [15].

In recent years, machine learning (ML) and deep learning have been developed to solve complex problems in various fields. Traditional machine learning methods are based on statistics and must extract features from the raw data first. One study attempted to evaluate Support Vector Machine analysis with 819 subjects in 2011 [16]. They chose nine Volumes of interest (VOIs) manually as features that are known to be affected in early AD. SVM was trained with those VOIs and certain filters, which were pruned by Random Forest (RF). The model showed 0.97 AUC (sensitivity 89%, specificity 94%) in distinguishing normal controls (NCs) from AD, and 0.92 AUC (sensitivity 89%, specificity 80%) in distinguishing NCs from MCI. Rather than manual feature extraction, Salvatore et al. introduced Principal Component Analysis (PCA) to extract features [17]. They presented a SVM-based classifier with 509 subjects, and extracted the features using PCA with preprocessed images. The model achieved 76% classification accuracy for NC vs. AD, and 72% for NC vs. MCI patients. They focused on interpreting the results and constructed a voxel-based pattern distribution map to identify meaningful features. Other studies have used deep learning methods, which are known to be good feature extractors and classifiers, simultaneously.

A recent study applied the Convolutional neural network (CNN) which is one of the popular deep learning methods [18]. In a test with 695 subjects’ MRI data, a CNN-based auto encoder model showed 86.6% classification accuracy for controls vs. AD, and 73.9% classification accuracy for controls vs. MCI.

However, the models mentioned above are somewhat limited in certain respects. First, manual feature selection requires a profound understanding of the disease and its cause/correlates, and a long and time-consuming analysis. Second, it is difficult to identify the major contributors (or biomarkers) to classification from deep learning models because of the models’ high complexity and architecture. Third, a single classifier may be less reliable. Thus, more robust, stable, and interpretable classifiers may be more suitable in classifying and understanding AD, given the high dimensionality and complexity of brain MRI features. One such classifier is the Random Forest (RF) model, which is an ensemble algorithm. This model has several advantages over other methods, such as the ability to manage highly non-linearly correlated data, robustness to noise, and a structure for efficient parallel processing [19]. Indeed, RF has shown good performance in various scientific fields [20,21,22,23]. However, despite its strength, this model is not investigated actively as a method to predict or understand AD. In the past decade, several studies have reported that RF demonstrates better performance than other methods. However, those studies evaluated RF with relatively small samples (*N* = 26 to 870 subjects). Thus, it is timely to investigate RF’s performance and compare it with other machine learning methods, including deep learning, with relatively larger datasets.

The aim of this study is to evaluate RF model and investigate its effectiveness in predicting AD with a relatively large data which was not used in literatures. Additionally, with the help of RF, we attempted to identify the meaningful brain areas which discriminates AD from normal control. To do so, we used 2250 subjects’ brain MRIs. Regional volumetrics were estimated, and we applied RF to classify NC, MCI, and AD patients. The results were compared with the conventional linear classifier SVM and two neural network models (Multi-Layer Perceptron and CNN). Finally, we investigated the input features based on their importance, which is one of the advantages that RF provides to identify AD’s promising biomarkers. As a result, we demonstrate that the three areas, hippocampus, amygdala, and inferior lateral ventricle, are promising biomarkers for AD.

The next sections are organized as follows. In Section 2, the ADNI data, data processing, and RF model for data analysis are explained. In Section 3, we present the results from various models and biomarker identification by RF. Then, the results and limitations are discussed in Section 4. Finally, we conclude this study in Section 5.

## 2. Materials and Methods

### 2.1. Brain MRI Data

The data used in this study were obtained from the Alzheimer’s Disease Neuroimaging Initiative (ADNI) database (adni.loni.usc.edu, accessed on 28 February 2021) [24]. The ADNI was launched in 2003 as a public-private partnership led by Principal Investigator Michael W. Weiner, MD. Its primary goal is to test whether serial magnetic resonance imaging (MRI), positron emission tomography (PET), other biological markers, and clinical and neuropsychological assessment can be combined to measure the progression from MCI to early AD. ADNI has three datasets, ADNI1 (2004–2009), ADNI2/GO (2010–2016), and ADNI3. We used part of the ADNI1 dataset with T1 weighted images. For current information, see www.adni-info.org (accessed on 28 February 2021).

The data included 2250 subjects with age, CDR, and MMSE scores. On average, 75-year-old subjects were examined who included 687 NC, 1094 MCIs, and 469 AD patients. The clinical dementia rating (CDR) is one of the representative scores used to assess recognition and social function [3], and is divided discretely into 0, 0.5, 1, 2, 3. NC subjects received 0, MCI subjects 0.5, and dementia subjects more than or equal to 1 according to their symptoms’ severity. Among dementia subjects, some have AD dementia and checked as amyloid positive through a positron emission tomography (PET) scan. The Mini-Mental State Examination (MMSE) is also a representative score used to assess various recognition states [4]. On the MMSE, NC subjects received from 30 to 24, MCI subjects from 23 to 18, and AD subjects from 17 to 0. The information on the data is summarized in Table 1.

**Figure 1:**
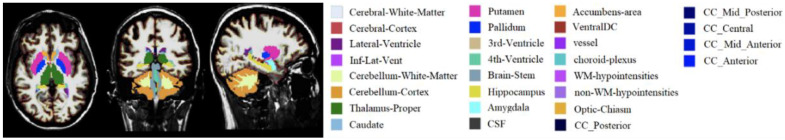
Brain segmentation provided by FreeSurfer software.

FreeSurfer (https://surfer.nmr.mgh.harvard.edu, accessed on 28 February 2021) is an open-source software used to process and analyze human brain MRI images [25]. We used FreeSurfer v. 6.0 with intel i9-9980XE CPU, which is run with OpenMP as the 8 threaded option. This software provides volumetric skull stripping, image registration, cortical segmentation, thickness estimation, longitudinal processing, visualization, and many more functions for brain MRI images. We segmented brain regions and obtained their volumetric information through FreeSurfer. An example of FreeSurfer’s segmentation result is shown in Figure 1. On average, we obtained four processed results from four datasets every four hours with parallel computing. Overall, 2250 data were generated over the 3~4 months of the study.

### 2.2. Feature Selection

Initially, we obtained 63 volumetric features from processing with FreeSurfer, as listed in Table 2. Then, a further feature selection procedure was applied to choose meaningful feature sets that may be beneficial for certain machine learning algorithms. Two steps were conducted in the feature selection procedure. First, we estimated the statistical significance of differences in all possible pairs of the three groups (NC, MCI, and AD). Three pairs were tested with Welch’s *t*-test for each feature (e.g., volume information), and 29 features that differed significantly (*p* < 0.05) in all comparisons (NC vs. MCI, MCI vs. AD, and NC vs. AD) were chosen as the meaningful feature set. These features are BrainSegNotVent, BrainSegNotVentSurf, VentricleChoroidVol, lhCortex, rhCortex, Cortex, lhCerebralWhiteMatter, rhCerebralWhiteMatter, CerebralWhiteMatter, SubCortGray, TotalGray, BrainSegVol-to-eTIV, lhSurfaceHoles, EstimatedTotalIntraCranialVol, Left-Lateral-Ventricle, Right-Lateral-Ventricle, Left-Inf-Lat-Vent, Right-Inf-Lat-Vent, Left-Putamen, Right-Putamen, 3rd-Ventricle, Left-Hippocampus, Right-Hippocampus, Left-Amygdala, Right-Amygdata, Left-Accumbens-area, Right-Accumbens-are, WM_hypointensities, and Optic-Chiasm.

Second, we identified features that showed a consistent increasing or decreasing pattern in the mean value from NC to MCI to AD. Thus, 6 features were removed from the 29 features, and finally 22 features were retained—VentricalChoroidVol, lhCortex, rhCortex, Cortex, SubCortGray, TotalGray, BrainSegBol-to-eTIV, lhSurfaceHoles, Left-Lateral-Ventricle, Right-Lateral-Ventricle, Left-Inf-Lat-Vent, Right-Inf-Lay-Vent, Left-Putamen, Right-Putamen, 3rd-Ventricle, Left-Hippocampus, Right-Hippocampus, Left-Amygdala, Right-Amygdala, Left-Accumbens-area, Right-Accumbens-area, and WM-hypointensities.

### 2.3. Random Forest Algorithm (RF)

The RF algorithm is a type of ensemble algorithm that consists of many Classification and Regression Trees (CART) [26]. These trees are trained with bootstrapped samples and the aggregated models’ results. This process, referred to as bagging, prevents the model from overfitting and generalizes well. As each tree grows, it sets its child nodes’ judgment to maximize the amount of newly acquired information. It can be represented by the Gini impurity, which is the same as the Gini index, and is calculated as follows:

Gini Impurity = 1 − ∑p_j_ (1 − p_j_) (1)

in which p_j_ denotes the probability that an element will be classified for a distinct class [27]. Each tree grows in a direction that minimizes the Gini impurity. All trees receive a dataset shuffled randomly and grow differently. These trees produce results with real data, and largely, the voted class is selected.

### 2.4. Classification Analysis

The three groups’ (NC, MCI, and AD) classification accuracy was estimated for each machine learning model. Every processing step was conducted in Python using the Scikit-learn [28] and Pytorch [29] libraries. For the RF models, the number of trees for bagging was set to 5000 which was manually set and large numbers considering related studies, and the RF classifier (RFC) and regressor (RFR) were constructed and compared. We also tested a conventional linear SVM and a non-linear SVM with a Radial Basis Function (RBF-SVM) kernel. The regularization parameter, ‘C’, in Scikit-learn was set to 1.0 for both SVMs, and the kernel coefficient, ‘gamma’, was set to 1/(num. of features * X.var()) for RBF-SVM which are the default values in Scikit-learn framework.

Two different neural network models were generated. MLP is a feedforward artificial neural network that consists of input-hidden-output layers. CNN is like MLP, but it includes convolution layers, some of which are connected sparsely or in part rather than fully. The MLP and CNN structures were designed as follows. MLP is constructed with one input layer, three hidden layers, and an output layer. CNN consists of two convolution layers and three fully connected layers with a kernel size of 4, stride 1, and no zero-padding settings. As the input data are one-dimensional, convolution layers were implemented with a one-dimensional convolution layer. 

**Figure 2:**
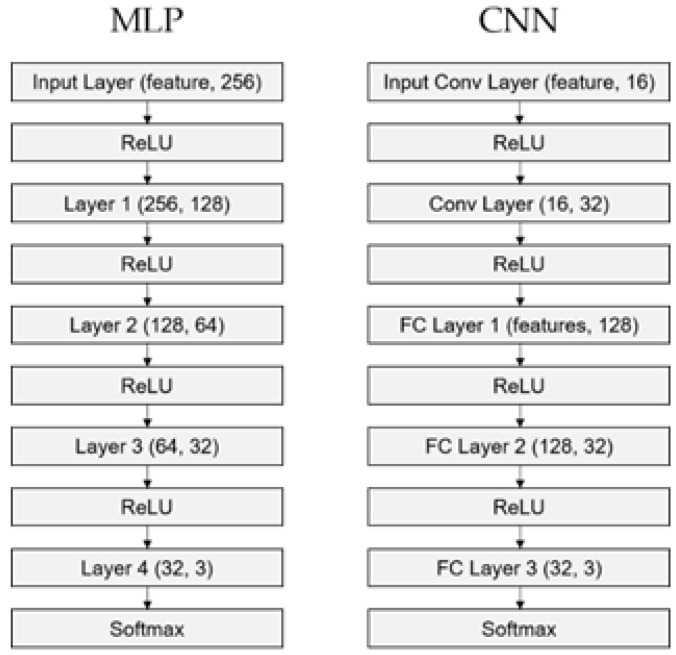
Structure of neural network models. MLP is the structure of the multi-layer perceptron model. The features in Input Layer were either 63, 29, or 22. CNN is the structure of the convolutional neural network model. The features in FC Layer 1 are either 1824(32 × 57), 736(32 × 23), or 512(32 × 16).

For these hyperparameters (number of hidden layers, kernel size, and stride), we tried to keep the same complexity between MLP and CNN. Thus, two models were designed with four hidden layers and the same structure of the last layer. After several simulations, other parameters were chosen empirically. The Rectified Linear Unit (ReLU) function and Softmax were used as activation functions between two consecutive layers and after the output layer, respectively. Loss was estimated based on Cross entropy, as it is used widely for multi-class classification. The Adam optimizer was used for backpropagation of loss, and early stopping was applied during training the MLP and CNN, which is activated when the testing loss is greater than the previous loss value more than 7 times consecutively. The MLP and CNN’s structures are presented in Figure 2.

The models’ classification accuracies were estimated with the cross-validation technique. The given feature sets were z-scored for normalization and corresponding labels were marked 0(NC), 1(MCI), and 2(AD). The normalized data were split into training and test sets and were fed into the cross-validation algorithm to produce a model based on the training data and test data’s classification accuracy. In addition, with the three conditions of the number of features (63, 29, and 22), we constructed various models repeatedly and generated their results. This procedure was repeated 100 times using 10 × 10-fold cross-validation, and finally, the mean accuracy and standard deviation were obtained for each model and feature set. Then additionally, Precision, Recall and F1-score of RF algorithm were calculated for further investigation.

### 2.5. RF-Based Biomarker Analysis

The benefit of using RF is that this algorithm provides the features’ importance, which is effective in identifying biomarkers. As the RF model selects more promising features for classification, the Gini index is an effective indicator when identifying biomarkers. Unlike the conventional approach, which selects the target biomarkers before the classifier model is constructed, RF-based biomarker identification is more useful, particularly when dealing with high-dimensional and non-linear data. In this study, we investigated the feature importance calculated as the decrease in node impurity (Gini Index), which consisted of 5000 decision trees. As mentioned before, we constructed the RF model with three kinds of features repeatedly and obtained the feature importance. Based on the chance values, which are 0.016, 0.035, and 0.046 for 63, 29, and 22 features respectively, the features that had a feature importance higher than the given threshold were selected, and the common features across the three conditions were identified as the final promising feature set. The volumetric values of the biomarkers identified were analyzed to determine the differences among NC, MCI, and AD, and statistical significance was evaluated with Welch’s *t*-test.

## 3. Results

### 3.1. Classification Accuracy

**Figure 3:**
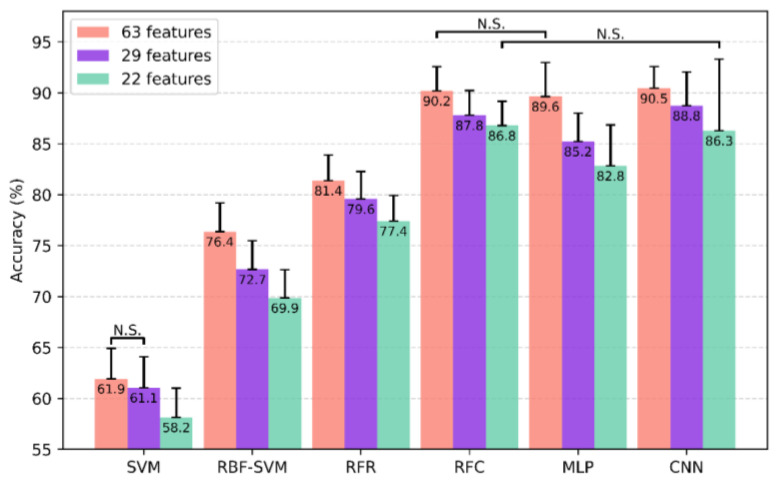
Various models’ classification accuracy with standard deviation. Pairs that did not differ significantly are marked N.S. (non-significant).

All models were trained with 63 features, 29 features, and 22 features, respectively, and tested with 10 × 10-fold cross-validation. The classification accuracies are presented in Figure 3. Every model showed the highest classification accuracy with more features, except for linear SVM, which showed no significant difference between the 63 and 29 feature sets. Indeed, linear SVM exhibited the lowest performance. Comparing the accuracy across models, RF (90.2% ± 2.4%), MLP (89.6% ± 3.3%), and CNN (90.5% ± 2.1%) with 63 features yielded relatively high accuracies near 90% for the three-class problem.

As the number of features decreased, the amount of information the data held also decreased. However, some models’ accuracies decreased only slightly, while others lost considerable accuracy. With the 22-feature set, CNN and MLP’s accuracy decreased by −4.6% and −7.6%, respectively, while RF showed a smaller decrease of −3.8%. Interestingly, the standard deviation increased from 2.1% to 7.0% in CNN, and from 3.3% to 4.0% in MLP. However, no change was observed in RF, 2.4% to 2.4%.

Precision, Recall, and F1-score are also important metrics in measuring performance. Table 3 presents those values calculated from RF model. As a result, 63 feature-set also shows the best performance over all cases. Interestingly, Precision (97.9%) which is positive predictive value, is higher than Recall (74.1%) and F1-scores (84.4%) for AD classification.

### 3.2. Biomarker Identification

RF provides feature importance information which is effective in identifying the meaningful brain areas. This means that features with higher feature importance are likely to better discriminate one condition from others. We attempted to investigate these scores to check which areas are promising in classification of three groups.

**Figure 4:**
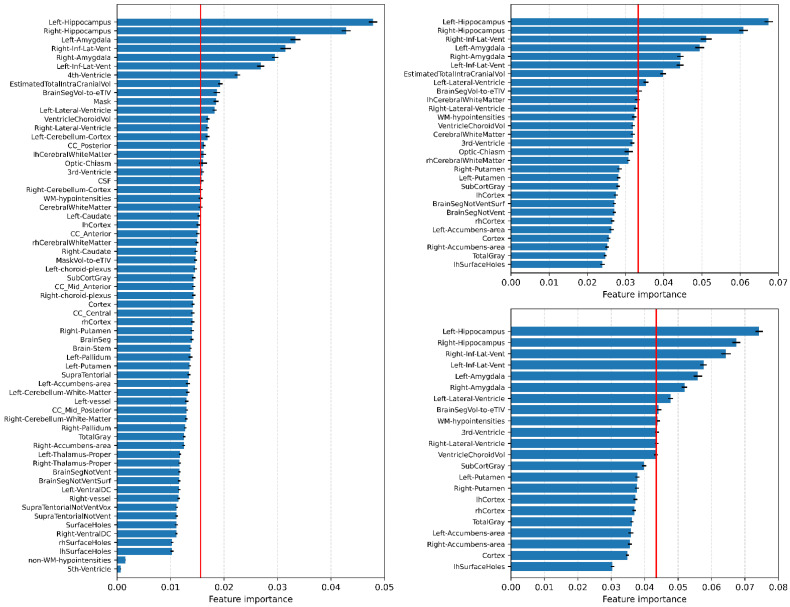
Feature importance obtained from RF classifier. Vertical red line is the chance level calculated. Feature importance is presented for 63, 29, and 22 feature groups.

**Figure 5:**
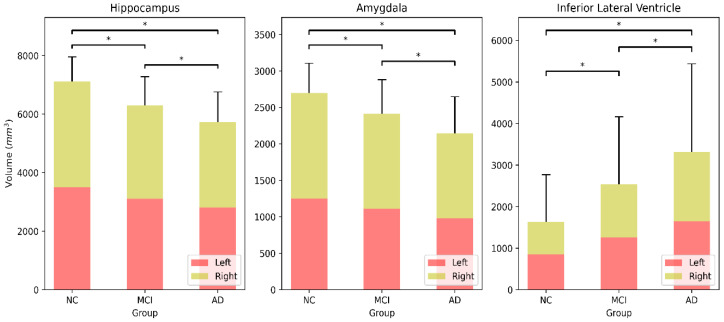
Regional volume of hippocampus, amygdala, and inferior lateral ventricle for NC, MCI, and AD groups. Statistical significance is marked with * (*p* < 0.05).

Feature importance values for each of the three feature groups were extracted and averaged from the RF classifier with 5000 decision trees, and the results are presented in Figure 4. We obtained the common areas in which the corresponding feature importance crossed the chance line (red vertical line in Figure 4). In the 63, 29, and 22 feature sets, 16, 8, and 7 features were identified, respectively. Among the 16 features, 4th-Ventricle and CC_Posterior showed no significant difference in any comparisons, and Mask and Left-Cerebellum-Cortex differed significantly in the NC vs. MCI and MCI vs. AD comparisons. Among eight features, EstimatedTotalIntraCranialVol showed no increasing or decreasing pattern in the mean value from NC to MCI to AD. As a result, six areas were identified; Left-Hippocampus, Right-Hippocampus, Left-Inf-Lat-Vent, Right-Inf-Lat-Vent, Left-Amygdala, Right-Amygdala. Combining the left and right sides, we identified three promising brain regions, the hippocampus, amygdala, and inferior lateral ventricle. The hippocampus and amygdala shrunk, while the inferior lateral ventricle enlarged as the symptoms worsened. This combined volumetric information’s statistical test results are shown in Figure 5, in which the three regions differed significantly in all comparisons.

**Figure 6:**
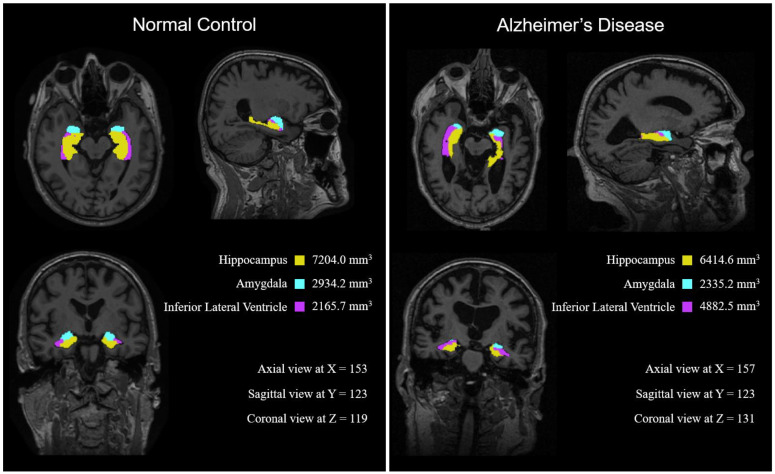
Two representative MRI images from the NC and AD groups. The three areas identified are marked as follows: Hippocampus: Yellow, Amygdala: Cyan, and Inferior Lateral Ventricle: Purple). Notably, the NC’s hippocampus and amygdala are larger and the inferior lateral ventricle is smaller than that of the AD subject.

Figure 6 shows MRI images of two representative subjects from the NC and AD groups, in which shrinkage of the hippocampus and amygdala and enlargement of the inferior lateral ventricle is observed clearly. The hippocampus shrunk from 7204 mm^3^ to 6414 mm^3^, the amygdala from 2934 mm^3^ to 2335 mm^3^, and the inferior lateral ventricle enlarged dramatically from 2165 mm^3^ to 4882 mm^3^.

## 4. Discussion

RF may be an effective tool or model in MRI analysis. In general, MRI comes in high dimensional data which may introduce a bias or produce less reliable results. RF is relatively robust to noise and complex data (e.g., high dimensional and highly correlated data). In addition, this model provides feature importance score which is useful in investigating biomarkers. However, RF has been applied in only several studies in the neuroimaging field for diagnostic classification of AD. Unfortunately, extant studies with RF have evaluated only relatively smaller samples [27] because of difficulty collecting medical brain MRI data. 

This study included a large number of subjects to create robust models for unclear data. As shown in Figure 3, the RF classifier achieved reliable accuracies with lower standard deviations not only compared to other studies [17,18], but also other neural network models. Identifying the disease’s biomarkers through learned models is as important as producing highly accurate models to classify or predict AD. Interpreting models’ judgment process can explain which models have been learned from data pools and detect which features are important to classify the disease. We evaluated feature importance values to ascertain biomarkers through the trained RF models shown in Figure 4.

The principal change in AD is the loss of neurons in the hippocampus and the amygdala’s atrophy [30,31]. The smaller the hippocampus and amygdala’s volumes, the more likely the patient is to be diagnosed with AD. As the hippocampus and amygdala are responsible for short- and long-term memory, these regions’ extreme shrinkage is the primary reason for memory loss, which is the main symptom of AD. Figure 4 shows that the high-ranked features related to AD’s progression are the hippocampus, amygdala, and inferior lateral ventricle. There may be a causal relation in which the hippocampus’s contraction enlarges the lateral ventricle [32], because the inferior lateral ventricle is a space in the cerebral hemisphere filled with fluid and the hippocampus lies adjacent to it. Figure 5 demonstrates these changes clearly and proves that the loss in the hippocampus and amygdala’s volume and enlargement of the inferior lateral ventricle are related strongly with AD’s progression.

We demonstrated that machine learning techniques distinguish AD patients from MCI and NC subjects well. In particular, RF, MLP, and CNN achieved approximately 90% classification accuracy. However, as we observed in Figure 4, RF showed robust performance with a smaller number of features. Further, given the change in the standard deviation from 63 features to 22 features, RF demonstrated the greatest stability. This may be because its internal structure uses a voting method from outputs from many decision trees. In this study, we used 5000 trees, which is a large number, and believe that this structure led to the RF model’s reliability. On the other hand, RFR show around 81% for 63 feature set which is lower than RFC, MLP and CNN. This low performance may be because of dataset. In this study, we did not use MRIs from the different stages of AD. However, we used MRIs of three distinct groups (NC, MCI and AD) because of the limited information in the ADNI data used in this study. Nonetheless, we think that 81% is somewhat of an interesting result, because this means that RFR can predict at least three stages from NC to MCI to AD. However, the well-defined data which represents disease stages of each patient, is necessary for solid conclusion. In the future, we aim to collect such data and design a model to predict the stages of disease progression.

On the other hand, there are new trends to explain the judgment of deep learning and other artificial intelligences that have been developed since 2017 [33], which are referred to as explainable AI (XAI) technology. Although deep learning methods’ performance is excellent, it is important to interpret and explain the way they ensure the results. Through this technology, we can understand what the deep learning model learned from the data. These insights are valuable for many research topics, and various topics use this technology [34]. Our research will allow us to determine the hidden communications between each part of the brain with AD’s onset if we can demonstrate that deep learning models perform perfectly. Relations in AD may exist among some of the 22 features that achieved high feature importance factors. Further, in various models, these features can serve as strong biomarkers to diagnose AD.

We used FreeSurfer in this study to segment brain regions. Thus, volume estimation’s quality may be less accurate because over- or under-estimation occurs in processing. Indeed, it has been reported that, at least in a pediatric case, the volume of the hippocampus and amygdala that FreeSurfer obtained may be inaccurate [35]. Although our data did not constitute a pediatric population, there is a slight chance that the automatic segmentation may introduce a bias or small error because the segmentation is not optimal. We believe that more accurate segmentation algorithms will improve the volume estimation’s accuracy and possibly increase the significance of the results. Another issue related to segmentation is that 63 features were evaluated in this study. |However, these may not be sufficient to represent the whole brain areas. For example, entorhinal cortex was not included and could not be evaluated by RF model. However, the area also undergoes atrophy such as hippocampus and amygdala during the disease progression [36,37,38]. Thus, more areas which are from an advanced and accurate brain segmentation algorithm, should be evaluated for identifying further promising biomarkers. 

## 5. Conclusions

In this study, we constructed an RF model with a large number of sub-trees (N = 5000) to classify NC, MCI, and AD with a large sample of brain MRI data (N = 2250). An RF model with three feature sets was evaluated and compared with other machine learning methods, including neural network models. As a result, we confirmed that the RF model had performance comparable to that of deep learning model, but was more robust and stable with fewer features. In addition, we identified three brain areas, the Hippocampus, amygdala, and inferior lateral ventricle that distinguished AD patients from NC’s best. In conclusion, RF is a powerful tool for classification and biomarker identification.

## Figures and Tables

**Table 1:** Demographic information for NC (normal controls), MCI (mild cognitive impairment), and AD (Alzheimer’s disease) groups (N = 2250).

	NC	MCI	AD
Subjects	687	1094	469
(Male, Female)	(357, 330)	(702, 392)	(249, 220)
Age	76.41 ± 5.07	75.42 ± 7.08	75.05 ± 7.60
CDR (Clinical Dementia Rating)	0.01 ± 0.13	0.51 ± 0.14	0.85 ± 0.41
(No. of subjects ^1^)	(687)	(1091)	(468)
MMSE (Mini-Mental State Exam)	29.07 ± 1.11	26.51 ± 2.62	22.42 ± 3.32
(No. of subjects ^1^)	(686)	(1090)	(468)

^1^ Some subjects do not have survey-based test result.

**Table 2:** Description of segmented parts of brain provided by FreeSurfer.

Name	Description	Name	Description
BrainSeg	Brain segmentation volume	Caudate	Volume of caudate
BrainSeg NotVent	Brain segmentation volume without ventricles	Putamen	Volume of putamen
BrainSeg NotVentSurf	Brain segmentation volume without ventricles from surf	Pallidum	Volume of pallidum
Ventricle ChoroidVol	Volume of ventricles and choroid plexus	3rd-Ventricle	Volume of 3rd-Ventricle
Cortex	Total cortical gray matter volume	4th-Ventricle	Volume of 4th-Ventricle
Cerebral WhiteMatter	Total cerebral white matter volume	5th-Ventricle	Volume of 5th Ventricle
SubCortGray	Subcortical gray matter volume	Brain-Stem	Volume of brainstem
TotalGray	Total gray matter volume	Hippocampus	Volume of hippocampus
SupraTentorial	Supratentorial volume	Amygdala	Volume of amygdala
SupraTentorial NotVent	Supratentorial volume without ventricles	CSF	Volume of cerebrospinal fluid
SupraTentorial NotVentVox	Supratentorial volume without ventricles voxel count	Accumbens-area	Volume of the nucleus accumbens
Mask	Mask (skull tripped) volume	VentralDC	Volume of ventral diencephalon
BrainSegVol-to-eTIV	Ratio of BrainSegVol to eTIV	vessel	Total volume of the brain vessel
MaskVol-to-eTIV	Ratio of MaskVol to eTIV	choroid-plexus	Volume of choroid plexus
SurfaceHoles	Total number of defect holes in surfaces prior to fixing	WM-hypointensities	Dark white matter on a T1-weighted image
EstimatedTotal IntraCraniaVol	Estimated total intracranial volume	non-WM-hypointensities	Dark gray matter on a T1-weighted image
Lateral-Ventricle	Lateral-Ventricle volume	Optic-Chiasm	Volume of optic chiasm
Inf-Lat-Vent	Inferior Lateral Ventricle volume	CC_Posterior	Volume of the corpus callosum in the posterior subcortical
Cerebellum-White-Matter	Total cerebellum white matter volume	CC_Central	Volume of the corpus callosum in the central subcortical
Cerebellum-Cortex	Cerebellum cortical gray matter volume	CC_Anterior	Volume of the corpus callosum in the anterior subcortical
Thalamus-Proper	Total Thalamus area volume		

**Table 3:** Precision, Recall, and F1-score for RF algorithm.

	Precision			Recall			F1-Score		
	NC	MCI	AD	NC	MCI	AD	NC	MCI	AD
63 features	92.9%	86.5%	97.9%	91.2%	96.5%	74.1%	92.0%	91.2%	84.4%
29 features	89.2%	84.9%	94.9%	89.1%	93.4%	73.3%	89.1%	88.9%	82.5%
22 features	88.3%	83.9%	93.9%	87.9%	93.3%	70.5%	88.0%	88.3%	80.3%

NC (normal controls), MCI (mild cognitive impairment), and AD (Alzheimer’s disease).

## Data Availability

The data presented in this study are openly available in Alzheimer’s Disease Neuroimaging Initiative at https://doi.org/10.1002/jmri.21049, accessed on 28 February 2021, reference number [24].
